# The Association between Fat Taste Sensitivity, Eating Habits, and Metabolic Health in Menopausal Women

**DOI:** 10.3390/nu13124506

**Published:** 2021-12-16

**Authors:** Aleksandra Skoczek-Rubińska, Agata Chmurzynska, Agata Muzsik-Kazimierska, Joanna Bajerska

**Affiliations:** Department of Human Nutrition and Dietetics, Poznań University of Life Sciences, Wojska Polskiego 31, 60-624 Poznan, Poland; aleksandra.skoczek-rubinska@up.poznan.pl (A.S.-R.); agata.chmurzynska@up.poznan.pl (A.C.); agata.muzsik@up.poznan.pl (A.M.-K.)

**Keywords:** fat taste sensitivity, fat taste thresholds, menopause, dietary habits, food intake, metabolic syndrome

## Abstract

The aim of our study was to evaluate the associations between sensitivity to fat taste, eating habits and BMI value in a sample of menopausal Polish women. In a population of 95 women, fat taste thresholds with oleic acid were determined, allowing us to classify each woman as a hypersensitive or hyposensitive taster. Eating habits were assessed using a validated KomPAN questionnaire for food frequency. Dietary intake was evaluated based on a food diary. Selected biochemical parameters were measured using a Konelab20i biochemical analyzer. Anthropometric parameters and blood pressure were also measured. Twenty-two menopausal women were classified as hyposensitive to fat taste and 73 as hypersensitive. The hyposensitive tasters were significantly older (*p* = 0.006), with the majority of them (92%) being postmenopausal (*p* < 0.001); this group had significantly higher BMI values (*p* < 0.001) and other adiposity indicators compared to their hypersensitive counterparts. The hyposensitive tasters had higher blood pressure (systolic blood pressure; SBP *p* = 0.030; diastolic blood pressure; DBP *p* = 0.003), glucose (*p* = 0.011) and triacylglycerols levels than the hypersensitive tasters (*p* = 0.031). Almost half of them had diagnosed metabolic syndrome. Daily eating occasions were associated with low oral fatty acid sensitivity, irrespective of age (*p* = 0.041) and BMI value (*p* = 0.028). There were also significant associations between frequency of consumption of meats and eggs, as well as snacks and fast foods and low oral fatty acid sensitivity before adjustment for potential confounders (both associations *p* < 0.05), which remained after adjustment for age (both associations *p* < 0.05), but not after adjustment for BMI. A multivariate logistic regression analysis showed that higher BMI value (*p* = 0.003), along with postmenopausal status (*p* = 0.003), were associated with low fat taste sensitivity irrespective of age and consumed percentage energy from fat. Postmenopausal status and BMI are associated with low fat taste sensitivity. Fat hyposensitivity may also play a role in eating habits, leading to increased eating occasions and favoring certain types of food. These eating habits may determine increased body weight and the occurrence of metabolic syndrome in mid-life women, especially those who have undergone menopause and have been exposed to the physiological changes which are conducive to these relationships.

## 1. Introduction

During the menopausal transition, clinical changes occur in body composition due to aging and decreasing ovarian function, with a reduction in ovarian hormones, mainly estrogens [1,2]. In ovariectomized mice, a significant increase in body weight and perturbations in related health outcomes were observed due to reduced energy expenditure and increased food consumption [3]. Studies of the link between food intake and body weight gain among menopausal women are rare and often inconclusive. However, a decline by 0.7% ± 0.1% per year in energy expenditure in people over 60 years of age was recently reported [4]. On the other hand, it has been suggested that the depletion of estrogens associated with the menopausal transition may lead to worsened perception of almost all tastes, as well as to weight gain, by affecting eating behavior [5,6]. For example, Delibasi et al. showed a significant reduction in sucrose perception and palatal sensitivity in a group of postmenopausal women [6]. A decreased perception of sucrose after menopause was also noted by Saluja et al. [7]. Animal studies have provided evidence for this hypothesis. The removal of ovaries from experimental mice led to diminished responses to linoleic acid and modified tastant-specific behaviors. This effect was reversed by exogenous administration of estradiol [8]. That study clearly showed that changes in the responsiveness of the taste system to fatty acid stimuli are intimately related to estrogenic perturbation in taste cells [8]. Curtis et al. also found that depletion of estrogen decreases salt and sucrose taste sensitivity, leading to increased consumption of products containing salt and sugar [9]. It has been suggested that fat taste sensitivity depends mostly on the environment, specifically, on fat intake. Relations between fat taste sensitivity, food intake, and body mass are not widely recognized [10]. Some studies have reported an inverse correlation between body mass index (BMI) and oral fat sensitivity [11,12,13]. However, a recent meta-analysis found that fat taste sensitivity does not contribute to or result from obesity [14]. Regarding the consumption of fat and fatty foods, studies have shown that people with lower sensitivity to fat taste tend to consume larger amounts of dietary fat [10,15], but some others failed to show such a relation [16]. In their review, Chmurzynska et al. suggested that there is interindividual variability in fat taste sensitivity, but that the methodological challenges of testing for fat sensitivity should also be taken into consideration when the relationship between body mass and fat taste sensitivity is being examined [17]. On this topic, Tucker et al. stated that repeated testing is required to properly assess the associations between fat taste and outcomes such as BMI and food intake [18].

To date no studies assessing the relationship between sensitivity to fat taste, fat intake, and body mass have been conducted on menopausal women. As mentioned, the menopausal transition may affect taste perception, and thus, through changes in fat detection, may affect food choices and metabolic outcomes. The aim of our study was thus to study the associations between fat taste sensitivity, eating habits, and adiposity indices in a sample of menopausal Polish women.

## 2. Materials and Methods

### 2.1. Study Design and Participants

A cross-sectional study was carried out at the Department of Human Nutrition and Dietetics, Poznań University of Life Sciences. Participants were recruited via advertisements in newspapers and social media between 2017 and 2020. One hundred and thirty-one Polish perimenopausal and postmenopausal women aged 43–69 years were recruited from the Wielkopolska region. Perimenopausal women and postmenopausal women up to six years after natural menopause, without hormone replacement therapy, were included in the study. The other inclusion criteria were that they did not participate in weight-loss programs and had no history of chronic systemic diseases such as type-2 diabetes, cardiovascular disease, or monogenic dyslipidemia. On account of the fat taste sensitivity test, additional exclusion criteria were applied: potential participants were excluded if they had used antibiotics in the three months before the beginning of the study, if they had used drugs affecting their ability to taste or the production of saliva; if they were currently smoking; if they had impaired taste function (e.g., due to chemotherapy), any oral or nasal diseases (cold or flu symptoms on the test days), allergies, intolerances, or aversion to milk or dairy products. During three consecutive visits, participants were assessed for fat taste sensitivity, eating habits, anthropometric, biomedical, and biochemical parameters. Other variables (age, menopausal status, age at last period, smoking history, and physical activity level) were also collected. Ultimately, the study included 95 women who met the inclusion criteria and showed willingness to participate in and complete the fat taste threshold test. Thirty-six women were excluded from the study due to not meeting the inclusion criteria, not completing the test, errors in the taste methodology or because of consultation with other participants during the fatty taste sensitivity test, as well as those classified as nontasters (participants who were unable to identify oleic acid, even at the highest concentration (20 mM)).

The sample size calculation was performed based on a previous study conducted by Haryono, Sprajcer, and Keast [11]. It was estimated that 95 participants were required based on alpha risk at 0.05 and beta risk at 0.2 (power 80%) to find a difference in oleic acid samples of different concentrations. The sample size was similar to those of other studies investigating oleic acid taste perception [10,12,19,20].

The study was conducted in accordance with the Declaration of Helsinki. The study protocol was reviewed and approved by the Poznań University of Medical Sciences (number 664/20). All participants provided their written informed consent prior to participating in the study.

### 2.2. Fat Taste Thresholds Test

Fat taste thresholds using oleic acid (C18:1) solutions were determined using triangle tests with ascending forced choice methodology (3-AFC), as described by Haryono et al. [11]. Oleic acid (Sigma Aldrich, natural, FCC, St. Louis, MO, USA) was added in increasing concentrations (0.02, 0.06, 1, 1.4, 2, 2.8, 3.8, 5, 6.4, 8, 9.8, 12, and 20 mM) to long-life, fat-free milk, based on the method proposed by Haryono et al. [11]. Textural differences were avoided by adding textural agents (gum Arabic and liquid paraffin). All tests were evaluated between 7 a.m. and 10 a.m. after an overnight fast to ensure that perception measurements were not influenced by hormonal fluctuations or by different levels of hunger. To prevent confounding nontaste sensory inputs, participants wore nose clips, and all tests were conducted under red light in separate booths in a sensory room at a temperature of around 20–22 °C. Participants were asked to rinse their mouths with deionized water both before the first sample as well as between sample sets.

Three randomly-ordered samples per set containing 30 mL vehicles prepared no more than 2 h prior to testing were presented at room temperature to each of the participants: these were an oleic acid sample solution with increasing concentration and two control samples. Participants were instructed to test and then choose or guess the sample that differed from the others. Correct identification of the C18:1 sample resulted in repeating the same sample set. Incorrect identification of the oleic acid sample resulted in being served a new sample with higher concentration of C18:1. Subjects correctly selecting the sample with oleic acid three times was taken as the detection threshold [21]. Based on previous papers, sensitivity to oleic acid was treated as a grouping variable and defined as “hypersensitive (high fat taste sensitivity)” when tasters had a fat taste threshold below 3.8 mM or “hyposensitive (low fat taste sensitivity)” when fat taste threshold was >3.8 mM [11,22]. Participants who did not correctly identify the oleic acid sample at the maximum concentration (20 mM) were defined as nontasters and were excluded from the study [19].

### 2.3. Anthropometric Measurements

Height was measured to the nearest 0.1 cm using a stadiometer (RadWag, Radom, Poland). Body weight was assessed to the nearest 0.1 kg after an overnight fast using the calibrated scale from a Bod Pod system (Cosmed, Albano Laziale, Italy). Body composition was measured using dual energy X-ray absorptiometry (DXA). Waist circumferences (WC) was measured using nonelastic tape placed horizontally just above the iliac crest with minimal respiration. The cutoff used for WC was that of European ethnicity (≥102 cm for men and ≥88 cm) [23]. Systolic and diastolic blood pressure (SBP and DBP, respectively) were measured using a sphygmomanometer on the day of the first meeting. Body Mass Index (BMI) was calculated by following formula: BMI = weight (kg)/[height (m)]^2^.

### 2.4. Biochemical Analysis

Blood samples were collected at the first meeting by qualified personnel. The serum, after collection, was obtained by centrifugation of venous blood clots at 1000 rpm for 10 min. The biological material was stored frozen at −80 °C until analysis. The levels of total cholesterol (T-C), high density lipoprotein cholesterol (HDL-C), low density lipoprotein cholesterol (LDL-C), triacylglycerols (TG), and glucose (GLU) were determined using an automated analyzer system (Konelab20i biochemical analyzer, ThermoElectron Corporation, Vantaa, Finland). Diagnosis of metabolic syndrome (MetS) was established according to the IDF criteria.

### 2.5. Physical Activity (PA) Estimation

PA was assessed using a short version of the International Physical Activity Questionnaire. The results were expressed as three PA levels: low (<600 metabolic equivalent [MET]/min/week), moderate (600–1499 MET/min/week), and high (≥1500 MET/min/week).

### 2.6. Dietary Assessment

Validated Dietary Habits and Nutrition Beliefs Questionnaire (KomPAN) was used to assess the frequency of consumption of 33 food items over the past year, with frequency consumption of items evaluated in six categories, i.e., from ‘never’ (1) to ‘a few times a day’ (6) [24]. For each food item, the frequency categories were converted to values reflecting daily consumption frequency (range: 0–2 times/day). For further analysis, food items from the FFQ were grouped into thirteen food categories: (1) meat and eggs, (2) fish, (3) dairy products, (4) grains, (5) fruits, (6) vegetables and legumes, (7) nuts and seeds, (8) fats, (9) sweets, (10) snacks and fast food, (11) sweetened beverages, (12) water, and (13) alcoholic beverages. The frequency of eating occasions was calculated by summing the frequency of consumption of main meals and snacks.

Besides the FFQ, participants also completed a four-day food diary. Before data collection, participants received detailed instructions on the type of food and drink consumed, time of food consumption, culinary techniques, and recipes (which should be recorded using household measures). The energy content of the daily food rations and intakes of lipids, carbohydrates, proteins, simple sugars, fatty acids, and dietary fiber were calculated using Dieta 6.0 software (National Institute of Public Health (PZH), National Research Institute, Warsaw, Poland).

### 2.7. Statistical Analysis

All the statistical analysis was performed using Statistica 13.0 software (TIBCO Software, Palo Alto, CA, USA), with the level of significance set at *p* < 0.05. The Shapiro–Wilk test was performed to verify the normality of the distribution of the variables and, as most data were nonnormally distributed, nonparametric statistical tests were used. Continuous data are presented as means and standard deviation (SD), and categorical data as *n*/%. Women assigned to the hyposensitive and hypersensitive for fat taste groups were compared for continuous and categorical variables using the Mann–Whitney U-test and the Chi2 test, respectively. A linear regression model unadjusted (model 1) adjusted for age (model 2) and additionally for BMI value (model 3) was built to test the relationship between: frequency of consumption of certain food groups, energy, and macronutrients intake (as dependent variables) and fat sensitivity threshold (as independent variable). The continuous dependent variables were normalized by a square root (SQR) transformations.

A stepwise logistic regression analysis with hyposensitive tasters as the dependent variable was calculated to predict factors associated with low fat teste sensitivity. We successively removed the least informative covariates from the model in a backward stepwise elimination procedure. The significant variables recognized between hypo- and hyper- sensitive fat tasters (e.g., BMI value, menopausal status and metabolic syndrome status) were therefore examined as independent variables in a multiple logistic regression. The model was adjusted to age and percentage energy obtained from the consumption of fat.

## 3. Results

The fat sensitivity test was carried out on 95 menopausal women, resulting in 73 women being classified as hypersensitive and 22 as hyposensitive. Three participants were not able to identify samples with the highest concentrations and were removed from the analysis.

Table 1 presents the distribution of study participants by fat taste sensitivity. Hyposensitive tasters were able to correctly distinguish samples at significantly higher oleic acid concentrations (*p* < 0.001) at 8.3 ± 5.5 mM than their hypersensitive counterparts (1.1 ± 0.9 mM). Hyposensitive women were significantly older, and the majority (92%) were postmenopausal (*p* < 0.001, Table 1). The mean values of BMI (*p* < 0.001), waist circumference (*p* < 0.001), percentage of fat mass (*p* = 0.006), and trunk fat mass (*p* = 0.004) were significantly higher in the hyposensitive women than in the hypersensitive women. There were also some biomedical and biochemical parameters that differed between the groups. The hyposensitive group had higher systolic (*p* = 0.030) and diastolic blood pressure (*p* = 0.003), as well as higher levels of fasting glucose (*p* = 0.011) and triacylglycerols (*p* = 0.031), than their hypersensitive counterparts. There were significant (*p* = 0.012) differences in the proportions of women with MetS. Almost half the hyposensitive women (49%) had diagnosed MetS, whereas this disease was diagnosed among 26% of hypersensitive women (Table 1).

Table 2 presents the relationships between frequency of consumption of certain food groups, energy, macronutrients intake and fat taste sensitivity adjusted for age and BMI. The association between eating occasions and low fat taste sensitivity was significant before adjustment for potential confounders (model 1), after adjustment for age (model 2) and after adjustment for BMI (model 3). There were also significant associations between frequency of consumption of meats, eggs, snacks and fast foods and low oral fatty acid sensitivity before adjustment for potential confounders (model 1), after adjustment for age (model 2), but not after adjustment for age and BMI (model 3, Table 2).

Women with higher BMI values who had undergone menopause were more likely to be fat hyposensitive tasters than women with lower BMI values (*p* = 0.003) and perimenopausal status (*p* = 0.003, Table 3).

## 4. Discussion

Our study has, for the first time, indicated that higher BMI values, along with postmenopausal status, irrespective of age and percentage of energy obtained from fat consumption, are associated with low fat taste sensitivity. One explanation for the association between BMI and sensitivity to fat taste is that decreased sensitivity to fat leads to an increased fat intake and weight gain. Liu et al., in their review, gave an example where both animals and humans that exhibit oral hyposensitivity to fatty acids were more likely to consume more fatty foods and experience weight gain than their hypersensitive counterparts [25]. Another explanation is that habituation to a high-fat diet leads to greater exposure to fatty foods due to the increased need to generate an appropriate oral response [26]. Chmurzynska et al. indicated that fat discrimination ability is not associated with the choice of high-fat food [16]. Alternatively, it may be that excess body weight itself modifies fat taste: obese participants displayed a reduction in type II cell markers, increased inflammation and reduced sonic hedgehog (SHH) signaling, which is critical in taste bud development and maintenance [27]. However, some studies have also reported a lack of association between BMI and fat taste sensitivity [28,29,30]. Finally, the results of a recent meta-analysis found that sensitivity to fat taste does not contribute to or result from obesity [14].

Our study showed a positive association between daily eating occasions and low oral fatty acid sensitivity, irrespective of age and BMI value. Additionally, there were also significant associations between frequency consumption of meats and eggs, as well snacks and fast foods, and low oral fatty acid sensitivity before adjustment for potential confounders, after adjustment for age, but not after adjustment for BMI. Martinez-Ruiz et al. showed that differences in oral fatty acid perception had an effect on preferences for the intake of foods rich in fat, like snacks and fast food [12]. Asano et al., in their study of younger (27-year-old) healthy individuals, observed that the oleic acid detection threshold was inversely associated with the degree of self-awareness of both spare eating (r = −0.466, *p* < 0.01) and satiety after food intake (r = −0.440, *p* < 0.01). The results of this study indicate that individuals who are hyposensitive to fat taste may fail to efficiently detect fat in the foods they eat, as well as receiving prolonged fat signals in their brain. In effect, such people do not stop their food intake earlier, leading to the excessive consumption [31]. Moreover, dietary fat has over twice the energy density of carbohydrates and proteins, but tends to be less satiating [32]. However, as mentioned earlier, environmental factors such as frequent consumption of fatty foods may be correlated with excess body weight, but not all support this relationship [33]. In addition, the relationship observed in our study between frequency of eating occasions, the consumption of meats, eggs, snacks and fast food and low oral fat sensitivity did not indicate the same relationship in daily energy intake, or in protein and fat consumption, expressed as a percentage of energy intake.

We also found that postmenopausal status, irrespective of age and percentage energy consumed from fat, was associated with lower sensitivity to fat taste. Recently, Dahir et al. indicated a significant role of estrogen in the peripheral taste system, specifically, in the modulation of cellular responses to fatty acids (fat taste) [8]. They observed that the presence of circulating estrogens increased apparent fat taste sensitivity among intact females as compared with their ovariectomized counterparts [8]. This suggests that as the years pass after menopause, following the loss of estrogen signaling, women are less responsive to the chemical cues in dietary fats, which may lead to eating more and putting on more weight than before menopause [8]. Menopausal changes are also associated with notable changes in levels of ghrelin [33]; the decreases in ghrelin observed in animal studies were also associated with decreased fat taste sensitivity [34,35,36]. On the other hand, Delilbasi et al. pointed out that the common oral discomfort of burning mouth syndrome (BMS) experienced by postmenopausal women may lead to chemosensory dysfunction and worsened food choices [6]. Our study also confirms that postmenopausal status is important for taste detection, and may be associated with changes in food intake.

This study has limitations that should be highlighted. First of all, the study sample was quite small (*n* = 95), though comparable with that of other studies of the association between fat taste sensitivity and BMI [10,21]. Distinguishing between oral fatty acid sensitivity may be hampered by differences in the textural properties of fatty acids and controls, or by the presence of oxidation products that can affect taste. However, as mentioned earlier, we adopted reliable methods to minimize the variation in texture and control fat oxidation [11], so any impact was likely negligible. One important aspect to note is that no single measure of the threshold is representative of taste function as a whole, as fats are complex stimuli that provide taste, olfactory, and textural cues, and are further influenced by their different physical states (liquid, solid, and semisolid). The validity of the present results could be improved by measuring fat on multiple occasions. One of the strengths of our research is that, to our knowledge, this is the first study to evaluate the associations between fat taste sensitivity, eating habits and adiposity measurements in a sample of menopausal women. Objective methods and calibrated devices set by one evaluator allowed high repeatability of the study results. Dietary intake was measured using a four-day food diary, and eating habits were measured using a validated food frequency questionnaire, allowing us to determine habitual consumption. The recommended fat taste sensitivity methodology for adults was applied in accordance with the author’s instructions [11], as used by other authors, thus allowing for direct comparisons to be made. Forced-choice paradigms are said to produce criterion-free, unbiased threshold estimates [19]. Furthermore, many studies, including our own, measured taste sensitivity on a single occasion only. As indicated, taste sensitivity may be affected by short-term caloric deprivation in both overweight and lean subjects, with lower thresholds of perception in the fasted state than in the satiated state. Thus, it could be suggested that some dietary restrictions may have an impact on taste sensitivity [37]. However, one of the eligibly criteria for our study was nonattendance in a weight loss program in the six months before the study began. Moreover, dietary restraint scores (data not shown) were similar between both groups of women.

## 5. Conclusions

These results suggest that postmenopausal status and higher BMI value, irrespective of age and percentage of energy obtained from fat, were associated with low fat taste sensitivity. Fat hyposensitivity may also play a significant role in eating habits, leading to an increased number eating occasions (irrespective of age and BMI) and increased frequency of consumption of meet and eggs, as well as snacks and fast food, irrespective of age, but not of BMI. These eating habits may determine increased body weight and the occurrence of MetS in mid-life women, especially those who have undergone menopause and experienced the physiological changes which are conducive to these relationships. More research is needed, in particular to investigate the possible link between the menopausal transition, brain regulation of appetite and satiety, and variability in oral fat sensitivity level, as well as the mechanism of the different preference degrees for highly processed foods between the oral fat hypersensitive and hyposensitive groups.

## Figures and Tables

**Table 1:** Distribution of study participants by fat taste sensitivity (mean ± SD).

Variables	Fat Hyposensitive *n* = 22	Fat Hypersensitive *n* = 73	*p*-Value
Fat taste perception level (mM)	8.3 ± 5.5	1.1 ± 0.9	<0.001
Age (year)	57.1 ± 5.7	53.9 ± 6.2	0.006
Perimenopausal (*n*/%)	2/8	28/38	<0.001
Postmenopausal (*n*/%)	20/92	45/62	
BMI (kg/m^2^)	30.5 ± 6.9	26.5 ± 5.3	<0.001
BMI distribution (*n*/%)			<0.001
BMI < 25 kg/m^2^	6/28	35/48	
25 kg/m^2^ ≤ BMI > 30 kg/m^2^	4/18	24/33	
BMI ≥ 30 kg/m^2^	12/54	14/19	
Waist circumference (cm)	106.3 ± 15.5	96.3 ± 11.6	<0.001
Fat mass (%)	42.8 ± 8.2	39.0 ± 6.6	0.006
Fat-free mass (kg)	44.8 ± 5.4	42.7 ± 58.8	0.060
Trunk fat mass (kg)	18.5 ± 7.8	14.4 ± 6.7	0.004
SBP (mmHg)	136.4 ± 20.0	128.1 ± 19.0	0.030
DBP (mmHg)	88.7 ± 9.9	83.0 ± 9.8	0.003
GLU (mg/dL)	97.1 ± 17.2	90.5 ± 10.8	0.011
TG (mg/dL)	145.1 ± 69.3	118.9 ± 58.4	0.031
T-C (mg/dL)	235.2 ± 41.2	220.4 ± 40.4	0.062
HDL-C (mg/dL)	60.1 ± 16.0	62.3 ± 14.7	0.463
LDL-C (mg/dL)	143.6 ± 33.9	135.1 ± 34.4	0.202
Presence of MetS (*n*/%)	11/49	19/26	0.012
PA low (*n*/%)	8/36	20/27	0.124
PA medium (*n*/%)	12/54	47/64	
PA high (*n*/%)	2/10	6/9	

Aberrations: BMI: body mass index; HDL-C: high density lipoprotein-cholesterol; LDL-C: low density lipoprotein cholesterol; DBP: diastolic blood pressure; FAT%: percentage of fat mass; FFM: fat-free mass; GLU: fasting glucose; MetS: metabolic syndrome; PA: physical activity; SBP: systolic blood pressure; T-C: total cholesterol; TG: triacylglycerols.

**Table 2:** Relationships between frequency of consumption of certain food groups, energy, macronutrients intake and fat taste sensitivity adjusted for age and BMI.

Dependent Variables	Low oral Fat Sensitivity Level (Independent Variable)			*p*-Value1	*p*-Value2	*p*-Value3
	Model 1 (Unadjusted)	Model 2 (Adjusted for Age)	Model 3 (Model 2 Plus BMI)			
Eating occasions (times/day)	0.20	0.19	0.21	0.027	0.041	0.028
Meat and eggs (times/day)	0.22	0.19	0.12	0.013	0.042	0.216
Fish (times/day)	−0.11	−0.05	−0.05	0.906	0.349	0.545
Milk and dairy products (times/day)	−0.02	−0.04	−0.04	0.819	0.707	0.875
Grains (times/day)	−0.01	−0.04	−0.01	0.910	0.633	0.923
Fruits (times/day)	0.06	0.06	0.13	0.522	0.555	0.188
Vegetables and legumes (times/day)	0.04	0.05	0.07	0.643	0.571	0.514
Nuts and seeds (times/day)	−0.02	0.01	0.04	0.801	0.919	0.685
Fats (times/day)	0.12	0.11	0.05	0.191	0.240	0.610
Sweets (times/day)						
Snacks and fast food (times/day)	0.18	0.20	0.18	0.041	0.032	0.063
Sugar-sweetened beverages (times/day)	−0.18	−0.16	−1.40	0.052	0.082	0.157
Alcoholic beverages (times/day)	0.002	0.02	0.01	0.982	0.872	0.915
Energy intake (kcal/day)	0.04	0.05	0.02	0.657	0.586	0.818
Percentage energy from protein	0.01	−0.02	−0.05	0.867	0.851	0.630
Percentage energy from fat	−0.15	−0.12	−0.09	0.099	0.172	0.346
Percentage energy from carbohydrates	0.13	0.12	0.18	0.130	0.187	0.217
Simple sugars (g/day)	0.05	0.06	0.04	0.587	0.514	0.647
Dietary fiber (g/day)	0.09	0.07	0.07	0.333	0.416	0.478

P1 unadjusted model, p2 model adjusted for age, p3 model adjusted for age and BMI.

**Table 3:** Associations between sensitivity to fat taste, BMI value and menopausal status.

Variables	*p*	OR (95% CI) *
Postmenopausal status	0.003	6.8 (1.9; 24.4)
BMI value	0.003	1.1 (1.0; 1.2)

* OR of being hyposensitive to fat taste using logistic regression. Adjusted for percentage energy from fat and age.

## Data Availability

The data presented in this study are available on request from the corresponding author.
